# Use of immigration status for coercive control in domestic violence protection orders

**DOI:** 10.3389/fsoc.2023.1146102

**Published:** 2023-04-28

**Authors:** Aisha Alsinai, Max Reygers, Lisa DiMascolo, Julie Kafka, Ali Rowhani-Rahbar, Avanti Adhia, Deirdre Bowen, Sandra Shanahan, Kimberly Dalve, Alice M. Ellyson

**Affiliations:** ^1^Firearm Injury & Policy Research Program, University of Washington, Seattle, WA, United States; ^2^Department of Pediatrics, University of Washington, Seattle, WA, United States; ^3^Department of Epidemiology, University of Washington, Seattle, WA, United States; ^4^School of Nursing, University of Washington, Seattle, WA, United States; ^5^School of Law, Seattle University, Seattle, WA, United States; ^6^Regional Domestic Violence Firearms Enforcement Unit, King County Prosecuting Attorney's Office, Seattle, WA, United States; ^7^Center for Child Health, Behavior, and Development, Seattle Children's Research Institute, Seattle, WA, United States

**Keywords:** immigration, coercion, domestic violence, legal protection, threats and attacks

## Abstract

In the context of domestic violence (DV), immigration-related circumstances can be exploited by an abuser to coerce and manipulate their partner. Using an intersectional structural framework, we examine how social structures overlaid with immigration-specific experiences operate to further enhance opportunities for abuse against immigrant women. We conducted a textual analysis to identify how socially constructed systems interact with a victim-survivor's immigration status to introduce more tools for abusers to engage in coercive control and/or acts of violence in a random sample of petitioners (i.e., victim-survivors) who were granted a Domestic Violence Protection Order (DVPO) in King County, WA (*n* = 3,579) from 2014–2016 and 2018–2020. We hand-reviewed textual petitioner narratives and identified *n* = 39 cases that discussed immigration-related circumstances and related acts of violence and coercion. These narratives included threats to contact authorities to interfere with an ongoing immigration process, deportation threats, and threats that would separate families. In many cases, petitioners indicated that immigration-related threats prevented them from leaving the violent partner, seeking help, or reporting the abuse. We also found mention of barriers for victims to receive protection and gain autonomy from further abuse including a lack of familiarity with US protections and laws, and restrictions on authorizations to work. These findings demonstrate that structurally created immigration-specific circumstances provide opportunities for threats and retaliation against victim-survivors by abusers and create barriers to seeking help initially. Policy should respond to anticipate these threats in the immigrant community and engage early responders (e.g., healthcare providers, law enforcement) to support victim-survivors from immigrant communities.

## Introduction

Over one-third of US women (36%) experience intimate partner violence (IPV), the most common form of domestic violence (DV) (Black et al., 2011). IPV describes any physical, sexual, or psychological abuse between current or former intimate partners (Breiding et al., 2015). While the prevalence of IPV among adults in the US is alarming, the prevalence among immigrants is even higher, ranging from 30%−60% (Sabina et al., 2014). More severe forms of IPV are disproportionately experienced by women compared to men (Black et al., 2011), and immigrant women are 1.9 times more likely to be victims of intimate partner femicide than US-born women (Frye et al., 2005). Research reveals that immigrant women may be particularly vulnerable to abuse because of compounding lived experiences of marginalization at the intersectional micro-level of gender, nationality, language, religion, race, ethnicity, and socioeconomic status (Crenshaw, 1991; Sokoloff and Dupont, 2006; Villalon, 2015) *and* at the macro-level through political, historical, and legal social structures related to immigration regulation that determine one's status and employability in the United States (Weber, 2010; Menjívar, 2011).

Because of social structural forces and systems of oppression that create social inequality, immigrant victim-survivors face specific barriers when seeking help (Malley-Morrison and Hines, 2007). Challenges reporting DV are exacerbated by limited access to social services, risk of deportation, family separation, distrust of systems, and limited English proficiency, which is further compounded with systemic discrimination based on race, ethnicity, gender, and sexuality (Lee, 2007; Weber, 2010; Rogerson, 2012; Reina and Lohman, 2015; Villalon, 2015; Abraham and Tastsoglou, 2016; Messing et al., 2017). These barriers structurally create opportunities for abusive partners to coerce and intimidate individuals who have immigrated or who are engaged in the US immigration process. They do so by leveraging these social structures to limit a victim-survivor's contact with family in the country-of-origin, prohibiting them from learning English, refusing to file papers to obtain legal status, threatening to contact immigration authorities, and restricting access to important documents (Raj and Silverman, 2002; Erez et al., 2009). These systems of oppression are then layered on to a victim-survivor's particular social context in several ways. Physical or emotional retaliation threats can prevent immigrant victim-survivors from seeking legal assistance (Voolma, 2018). Sometimes, cultural norms in immigrant communities dictate that DV should be handled informally within family and community circles without involving legal authorities. As a result, there may be socio-cultural ramifications for reporting DV in immigrant communities, like stigmatization and shame and family retaliation (Critelli and Yalim, 2020).

Stereotypes, racism, and other institutionalized forms of oppression manifest in multiple, nuanced ways to marginalize and disadvantage immigrant women who are victim-survivors of DV. For example, some people in the legal system may assume that certain immigrant or racial communities are violent, and therefore that immigrant women are rarely innocent or credible victims (Burman and Chantler, 2005). The use of a variety social institutions to stoke public xenophobic sentiments in the recent decade likely exacerbated hesitation from immigrant community members to engage the legal system for protection from abusive partners. In 2021, customs and border patrol arrests reached a record high. Increased negative media attention on immigration issues intensified the general public's hostility toward immigrants (Conzo et al., 2021). Finally, social media is used effectively to incite anti-immigration attitudes (Ekman, 2019).

The tactics of coercive control thrive under these circumstances. Coercive control is a pattern of behavior used to limit another person's autonomy and liberty, often involving a mixture of physical, emotional, and/or psychological harm (Stark, 2007, 2009). Extensive research demonstrates that coercive control relies on structures of oppression that invoke sexism, racism, and xenophobia (Douglas, 2021). Abusers adjust their strategies of coercive control, testing tactics that work best to control others at different points in time and under different circumstances; victim-survivors often point to these tactics as the worst forms of abuse (Douglas, 2021). The use of immigration-related circumstances may be a particularly effective form of coercive control because of the use of legitimate underlying societal structures that reify various systems of oppression (Crenshaw, 1991).

The goal of this study was to describe the lived experiences of DV victim-survivors who reported immigration-related circumstances when petitioning for a domestic violence protection order (DVPO) in King County, WA. A DVPO is a civil action that provides specific legal protections to petitioners, or victim-survivors, of DV. These protections often include things like no contact, surrender of dangerous weapons, and other prohibitions that can be enforced criminally if the respondent/abusive partner, violates. Findings from this study will inform policy and applied practice for legal advocates, attorneys, judicial officers, batterers' intervention providers and other practitioners by highlighting immigration-specific threats and retaliation that need to be addressed to serve victim-survivors who experience immigration-related coercive control.

### Immigration-related DV policies and protections

Federal legislation in the US provides protections for victim-survivors who are immigrants under the Violence Against Women Act (VAWA) as well as protections for victims of crimes (e.g., the “U” visa and “T” visa both created under the Victims of Trafficking and Violence Prevention Act (2000), Pub. L. No. 106-386, 114 Stat. 1464–1548). Both the “U” and “T” visa processes require engagement and ongoing cooperation with the criminal legal system. In 1994, the United States congress enacted the Violence Against Women Act (VAWA) to address DV, and over the last several decades VAWA has been reauthorized many times (2000, 2005, 2013). Most relevant to the current study, VAWA provides immigrants the opportunity to self-petition to maintain residence in the US after experiencing DV. Victim-survivors must meet very specific legal thresholds to obtain protection under all of these mechanisms, and these laws include prejudiced limitations that may not serve victim-survivors (Olivares, 2014). Further, these immigration-related protections may not address all ways abusers use the immigration system as part of coercive control. For example, none of these programs protect a family member of a victim-survivor whom an abuser may threaten to report to immigration as part of coercive control.

### Protections from coercive control

More recently, a recognition that focusing laws only on physical violence (i.e., “discrete, injurious assaults”) was too narrow prompted governing bodies to better codify recognition of coercive control in existing DV civil and criminal law (Stark and Hester, 2019). Some US states have expanded their definitions of DV to include coercive control, including WA (RCW 7.105.010) effective July 2023. There has been robust debate around these changes, including concerns about tangibility and reducing the effectiveness of addressing current forms of DV already codified in law like physical and sexual assault (Fitz-Gibbon and Walklate, 2017; Walby and Towers, 2017). Given its new and limited adoption in the law both internationally and in the US, rich qualitative research and robust quantitative evaluations are needed to determine how to balance the need to address addressing coercive control in the legal system (Stark and Hester, 2019). Importantly, these changes may differentially impact protection from abusers for certain victim-survivors, particularly those most impacted by social entrapment related to immigration-related circumstances (Ptacek, 1999).

## Method

### Data source

This study used a sub-sample of petitions from a larger study of a random sample of granted DVPOs from 2014–2016 and 2018–2020 in King County, WA (*n* = 3,579). In the parent study, three coders (AA, MR, LD) abstracted details from DVPO petitions and other court documents which are public record. This observational study was approved by the University of Washington Institutional Review Board after expedited review. The key fields of interest from DVPO petitions for this study include the victim's narrative about their experiences of abuse and DV. The DVPO application form asks petitioners to provide a statement describing specific experiences of violence as well as fears and threats of violence (e.g., incidents of violence where they were afraid of injury or where the respondent threatened to harm or kill them, violence or threats toward children; stalking; the use of firearms, weapons, or objects to threaten or harm them). Petitioners then provide a written account of the abuse, including what happened, where, who was involved, and when. These petitioner narratives have been used in prior research (Fitzgerald and Douglas, 2020; Redding et al., 2023), and they represent a unique form of textual data. While these narratives can provide rich insight into victim experiences, they are written to serve a very specific purpose; to describe abuse in a succinct and compelling manner so that a judge can decide whether to grant a DVPO.

### Data analysis

While abstracting case files for the parent study, our team read petitioner narratives, accompanying police reports, and other court documents. Cases were evenly divided among abstractors (AA, MR, LD) who independently reviewed all available text. We met regularly and debriefed the abstraction process. Our interest in immigration-related content from the DVPO case files emerged after encountering case narratives where the petitioner, their family, or their intimate partner was described as having migrated from another country to the US; feared expulsion from the US to the country from which they immigrated; or reported that their partner used immigration status to coerce, manipulate, or exploit them. We recorded cases with immigration-related circumstances as a binary yes/no.

After we identified the subset of cases that mentioned immigration-related circumstances (n=39), each coder separately extracted relevant, immigration-related quotes. Our team of coders (AA, MR, LD) and the study lead (AE), met as a group, reviewed all extracted quotes, and generated a list of codes and definitions. These inductive codes included “acts of violence,” “coercive control,” “deportation,” “ongoing immigration process,” “authorization to work,” and “familiarity with US protections and laws.” We applied these codes to the extracted text on immigration-related circumstances through group discussion and by consensus. We debriefed the process as a team and analyzed the data by organizing quotes in visual matrices based on the applied inductive codes (Sandelowski, 1995; Averill, 2002). We noticed that most mentions of immigration status were described in the context of coercive control. Through continued group discussion, we characterized the following ways that immigration status was exploited in the context of DV: threats of deportation, threats to separate families, (un)familiarity with US protection of laws, and authorization to work. The lead author (AA) wrote up these results which were iteratively reviewed and refined through conversations with co-authors.

### Reflexivity statement

Researchers' lived experience and subjectivity are a valuable resource for knowledge generation in qualitative data analysis; findings are actively created through interpretative engagement with the text and rich discussion within the research team (Braun and Clarke, 2021). As such, we foreground the results of our study with an acknowledgment of the positionality of the research team. Our interdisciplinary team included researchers and practitioners who provide legal advocacy to DV petitioners, represent petitioners and families in King County, and coordinate with law enforcement and the prosecuting attorney's office to provide robust safety planning when a DVPO is filed. The lead author immigrated to the US, worked as a medical interpreter, and advocates and interprets for her community. At the time of data analysis, most authors were affiliated with academic institutions as research staff, post-doctoral fellows, and early, middle-, and senior-career faculty in Schools of Medicine, Public Health, and Law.

## Results

We found that respondents (i.e., abusive partners) often used immigration-related factors as part of coercive threats and acts of violence that then created barriers for petitioners to obtain protection and avoid further abuse. These behaviors resulted in keeping petitioners from leaving the violent partner, seeking help, reporting to police, or having agency in their life. Almost all of the respondents reported as using immigration-related circumstances as part of coercive threats and abuse were men (97.4%; 38 of 39), a large proportion were Hispanic or Latino/a/x (43.6%; 17 of 39) or White (30.8%; 12 of 39), and a smaller proportion were Black (10.3%; 4 of 39), Asian (7.7%; 3 of 39), Native Hawaiian or Pacific Islander (2.6%, 1 of 39), or Middle Eastern or North African (2.6%, 1 of 39). Information on respondent race/ethnicity was unknown for *n* = 1 respondent. Neither gender nor race/ethnicity were self-reported by the respondent; they were assessed and recorded on the granted Order by a judicial officer, an attorney, or an advocate working with the petitioner. The average age of these respondents was 37.2 years [SD = 9.3, Median = 35.6, IQR = (30.2–43.3)]. The average age of petitioners reporting these experiences was 32.8 years [SD = 6.4, Median = 32.5, IQR = (28.2–36.6)], but information on petitioner gender and race/ethnicity was not available to our team for review. Information was also unavailable about the petitioner's country of origin or legal status in the US.

### Ongoing immigration process and threats of deportation

The ongoing immigration process for some petitioners, and sometimes for their loved ones, made petitioners reluctant to report to police and/or seek legal remedies. One petitioner stated, “He would touch me all over my body, and when I tried to say no, he would threaten to call immigration on me.” The respondent exploited the petitioner's immigration status to get them to comply with their demands even when the petitioner advocated for themselves. In another case, a respondent fabricated details he intended to use as part of immigration-related threats (Table 1). Several petitioners reported that the respondent directly threatened them with deportation. A petitioner stated the respondent would threaten, “If you don't have a kid with me, I will kick your butt back to Vietnam.” Deportation threats can impact the petitioner and individuals connected to the petitioner who are enmeshed in an ongoing immigration process (Table 1).

**Table 1:** Immigration-related themes and illustrative quotes.

**Immigration-related themes**	**Source**	**Illustrative quotes from petitioner narratives and police reports**
Ongoing Immigration Process and Threats of Deportation	Petitioners	“He would touch me all over my body, and when I tried to say no, he would threaten to call immigration on me, or hit me until I agreed” “I threatened to call the police, but he told me that he would frustrate my immigration case. So, I was too scared to call” “He told me before if you won't have sex with me, I am not going to process your green card” “A fair amount of his harassment toward me centers on my immigration status, which causes me severe emotional distress. He consistently accused me of engaging in criminal activities and threatens to tell immigration authorities about the alleged criminal activities. For example, he threatens to tell immigration authorities that my July 2019 marriage is a ‘sham' marriage, which is patently false” “Before I got my green card, my husband would hold onto all of my immigration papers” & “If you don't have a kid with me, I will kick your butt back to Vietnam” “The day we met, he proposed me to leave my partner and come back with him. When I said no, he threatened to call immigration on my partner… I told him this is ridiculous because it will hurt me as well because I am undocumented as well” & “If I did not do what he wants, then he will send immigration” “At the time, my child's birth father was undocumented. When the respondent found out, she told me that she was going to report my child's birth father to immigration. I was appalled”
Threats to separate families	Petitioners and Police report	“During their relationship, the respondent would physically and sexually assault her (petitioner), but she was too afraid to report this to the police as the respondent would threaten to have her deported and have the children taken away.”—Police report “My husband is constantly threatening me that if I ever call the police, he would call immigration services and have me deported and never see my kids again.”—Petitioner “[The respondent is] always threatening to call immigration on me because I am Mexican, making me too scared to do anything because he would say that he had a right over our daughter.”—Petitioner
Familiarity with US protections or laws	Petitioners	“At one point, the respondent got his mom to threaten me in 2013, and his mom said if I tried to leave [the relationship with the respondent] she would take the girls and have me deported. I am living here lawfully, but I was naïve and didn't understand the laws so these threats scared me” “He took advantage of the fact that I do not understand the rules and protections of this country”
Authorization to work	Petitioner	“He also called my workplace to tell them if they didn't fire me, he was going to call immigration on them and have all the workers arrested. One night my manager called me in and told me that they have to let me go because of the threats the respondent was making”

### Threats to separate families

Petitioners expressed that the respondents sometimes threatened to have them deported and separated from their children. The threats made them fearful and prevented them from seeking help. One petitioner said the respondent was “always threatening to call immigration on me because I am Mexican, making me too scared to do anything because he would say that he had a right over our daughter.” Respondents used threats to separate petitioners from their children with other forms of coercion and violence like derogatory language, physical and sexual violence, and threats to kill the petitioner. The threats of separation were paired with aggression toward children (e.g., yelling/screaming at them, hitting them, driving recklessly with them in the car, taking them away) that intensified the intimidation and impact of these threats.

### Familiarity with US protections or laws

Respondents used the petitioner's lack of familiarity with US protections and laws to instill fear and force the petitioners to comply with the respondent's requests. One petitioner stated, “At one point, the respondent got his mom to threaten me in 2013, and his mom said if I tried to leave [the relationship with the respondent] she would take the girls and have me deported. I am living here lawfully, but I was naïve and didn't understand the laws so these threats scared me.” Despite the petitioner lawfully residing as an immigrant in the US, the respondent used the petitioner's lack of knowledge of the US immigration system and laws to maintain control.

### Authorization to work

Another immigration-related tactic used to exploit petitioners was employment. One petitioner indicated, “He also called my workplace to tell them if they didn't fire me, he was going to call immigration on them and have all the workers arrested. One night my manager called me in and told me that they have to let me go because of the threats the respondent was making.” Causing a partner to lose their job is a form of economic abuse. It can lead to increased financial dependence on the respondent while socially isolating petitioners from their colleagues.

## Discussion

Consistent with prior research, we found that some DVPO petitioners reported respondents leveraged existing social institutions to further their power and control over victim-survivors (Reina and Lohman, 2015). Abusers relied on the immigration regulation system to exploit immigrant petitioners' safety. These forms included threats of deportation and family separation. These threats were effective because respondents knew to exploit petitioners' lack of familiarity with US protections and laws. Moreover, the immigration system, in combination with employment regulations results in restrictions on work authorizations, providing respondents even more power over petitioners. Again, respondents layered the weaponization of legitimate social systems over petitioners with particular social contexts, whether it be family relationships, isolation, economic vulnerability, or shared children. The weaponization of structural institutions like the immigration, legal, and employment system combined with the unique social and historical context of the victim-survivor were then combined with universal DV tactics (e.g., intimidation, economic abuse, threats against children) to control and exert power over petitioners, consistent with prior literature in the United States (Ammar et al., 2012; Messing et al., 2017; Njie-Carr et al., 2021). The findings specific to immigration-related circumstances have unique and important implications for policy.

We identified numerous immigration-related factors that involve legitimate social structures leveraged by abusers that ultimately influenced petitioner decision-making, including fears that an abuser may retaliate by jeopardizing their, or their family's or co-workers' status in the United States. Providing multiple culturally-appropriate, safe options for immigrant petitioners to report abuse could significantly improve help-seeking efforts among immigrant victim-survivors. For example, training law enforcement on the concerns immigrants have with the current DV system is a promising approach. One intervention that can potentially help to overcome immigration-related threats from DV respondents is to implement educational and outreach efforts in partnership with immigrant communities (Reina and Lohman, 2015). While social structures can be weaponized by abusive partners, they can also serve as a force for good by providing education on the laws and what is possible for employment, immigration, and deportation when an individual is experiencing DV. In addition, the Americans for Immigrant Justice Lucha Program offers free legal assistance to help immigrant victim-survivors obtain immigration relief. This program recognizes that victim-survivors and their loved ones in immigrant communities face specific barriers (e.g., limitations to work, opening a bank account, or getting a driver's license) that make it challenging to leave an abusive relationship. Local and national programs like the Lucha Program should be widely implemented and encouraged in advocacy, social services, and community settings. Because language barriers also provide an opportunity for abusive behavior, the legal system should offer more language options to increase access to legal help at all points of the process—from 911 operators to responding law enforcement officers to victim advocates to court interpreters. Additionally, employers who hire immigrant communities should be given training and information to distribute to employees about their rights in DV situations.

Our study showed that violence against women, and in particular against immigrant victim-survivors, comes from a complex interconnected system built on social structures, systems of oppression, and individualized social context. An abusive partner exploits all these sources to create fears of deportation and family separation. Actors in social structures that come into contact with immigrant community members should be aware of how they enhance systems of oppression (sometimes unwittingly) in ways that allow abusers to weaponize them against victims. It is also important for judges to be aware of these specific coercive tactics so they can be sensitive to these issues during DVPO case proceedings and provide legal protections or relief, when warranted clarity. For example, sometimes a petitioners' only available method of communication with advocates, lawyers, and judges may be an English-speaking abuser manipulating the narrative (Lee, 2007). If judges can recognize this situation and obtain appropriate interpretive supports, some of these problems could be mitigated. Thus, additional judicial training and awareness of these dynamics may be an important. Prior work finds that judges' implicit biases may also be exacerbated for petitioners from immigrant communities and may play into a perpetrator's narrative when petitioners from immigrant communities do access legal systems (Reina and Lohman, 2015; Espino-Piepp, 2018). These gender- and immigration-based institutional implicit biases include assumptions that victim-survivors lie about abuse to get a “leg up” in custody disputes, a baseline belief that abuse is exaggerated, or petitioners may be seeking legal protections from deportation by claiming abuse (Perrin, 2017). These biases are a result of the layered systems of oppression from social structures that are weaponized by abusers and then experienced by victim-survivors when they do engage the system.

Our findings suggest that limited US protections or laws may serve to amplify an abuser's coercive tactics to control their partners. In particular, victim-survivors often described immigration-related threats as a part of coercive control, but this form of abuse alone would not have constituted DV during our study period based on the official legal definition in WA. WA's expansion of the definition of coercive control may help address these abuse tactics. For example, an abuser contacting immigration about the victim-survivor or a new partner could now legally constitute harassment and coercion. Our findings are also consistent with other work showing that limited knowledge of the US legal system, lack of awareness of existing advocacy or social services, and limited English proficiency can prevent immigrant victim-survivors from utilizing the available legal resources that prevent them from getting deported (Reina and Lohman, 2015; Okeke-Ihejirika et al., 2020). More research is needed to understand whether these legal changes and increasing awareness about immigration protection and legal resources may help reduce DV perpetrators' opportunities to exploit immigrant petitioners' safety.

### Limitations

The cases we identified may not include all cases where immigration, deportation, or legal status were exploited as part of ongoing DVPO. Petitioners were not specifically prompted on the petition to discuss immigration-related concerns. Petitioners may be hesitant to share information about their immigration status that could potentially be used against them, as DVPO petitions are publicly available documents in WA and are formally served to DVPO respondents as part of the routine civil process. Importantly, we were unable to describe the experiences of petitioners who were not granted a DVPO (e.g., petitions that were denied, dismissed, or withdrawn). In addition, given that we used secondary, administrative data, we could not probe by asking follow-up questions about petitioner experiences. There may be many more petitioners who were reluctant to disclose these details because their immigration status was more precarious or because they did not know this was information they could/should include. We were not able to examine the intersectionality of immigration-related coercive control with racism, sexism, or other systems of oppression because demographic information on petitioners and victim-survivors (e.g., race/ethnicity, gender, sexual orientation) was not available in the court records we accessed. Further, the textual data provided on immigration-related circumstances was brief and limited. Given the unique nature of this data source and its high credibility (i.e., all DVPO requests in our sample were granted), we decided it was still important to share these findings which can now be considered for future research and practice. Lastly, we were not able to examine differences in violence related to types of immigration (e.g., asylum, refugee, student visa) because there are no details about citizenship or immigration status in the petition. More work is needed to understand how immigration systems can best recognize the abuse experiences of victim-survivors and work to address the unique needs of DV petitioners from immigrant communities.

## Conclusion

This study provides evidence that some DVPO petitioners' immigration-related circumstances are subject to exploitation by perpetrators of DV. Some DVPO respondents use petitioners' immigration status (real or perceived) to coerce and control them. The US immigration system as designed enables this abuse. In a system that deems some immigrants “illegal,” obtaining legal protection from DV becomes challenging. Revisiting legal policies is highly recommended to address the threats immigrant petitioners experience. Immigration-specific threats and forms of retaliation should be recognized by advocates and legal practitioners to address the specific needs of DVPO petitioners who interact with the immigration system.

## Data availability statement

The datasets abstracted and analyzed for this study are considered human subjects data, so they are not publicly available. De-identified study data and abstraction materials may be available upon request to interested researchers.

## Ethics statement

This observational study was approved by the University of Washington Institutional Review Board after expedited review. Written informed consent for participation was not required for this study in accordance with the national legislation and the institutional requirements.

## Author contributions

AAl, MR, LD, and AE conceived and designed the study and conducted qualitative analysis. AAl, MR, and LD conducted case review and coding. AAl and AE drafted the manuscript. AAl, AE, JK, AAd, and DB revised the manuscript. All authors contributed to the understanding and interpretation of qualitative data, critical revision of the article for important intellectual content, and approved the final manuscript as submitted.
